# Emerging Advances of Non-coding RNAs and Competitive Endogenous RNA Regulatory Networks in Asthma

**DOI:** 10.1080/21655979.2021.1981796

**Published:** 2021-10-11

**Authors:** Xiaoxu Wang, Hui Chen, Jingjing Liu, Linlin Gai, Xinyi Yan, Zhiliang Guo, Fengxia Liu

**Affiliations:** a Clinical Medicine College, Weifang Medical University, Weifang China; b Department of Allergy, The First Affiliated Hospital of Weifang Medical University/ Weifang People’s Hospital, Weifang China; c Department of Central Laboratory, The First Affiliated Hospital of Weifang Medical University/Weifang People’s Hospital, Weifang China; d Department of Spine Surgery, The 80th Group Army Hospital of Chinese PLA, Weifang China

**Keywords:** Asthma, non-coding RNAs, competing endogenous RNAs, pathogenesis, biomarker

## Abstract

Asthma is a chronic inflammatory disease characterized by airway remodeling and bronchial hyperresponsiveness. A variety of effector cells and cytokines jointly stimulate the occurrence of inflammatory response in asthma. Although the pathogenesis of asthma is not entirely clear, the possible roles of non-coding RNAs (ncRNAs) have been recently demonstrated. NcRNAs are non-protein-coding RNA molecules, such as circular RNAs (circRNAs), long non-coding RNAs (lncRNAs) and microRNAs (miRNAs), which are involved in the regulation of a variety of biological processes. Mounting studies have shown that ncRNAs play pivotal roles in the occurrence and progression of asthma via competing endogenous RNA (ceRNA) regulatory networks. However, the specific mechanism and clinical application of ncRNAs and ceRNA regulatory networks in asthma have not been fully elucidated, which are worthy of further investigation. This paper comprehensively summarized the current progress on the roles of miRNAs, lncRNAs, circRNAs, and ceRNA regulatory networks in asthma, which can provide a better understanding for the disease pathogenesis and is helpful for identifying novel biomarkers for asthma.

## 1. Introduction

**Figure 1.**
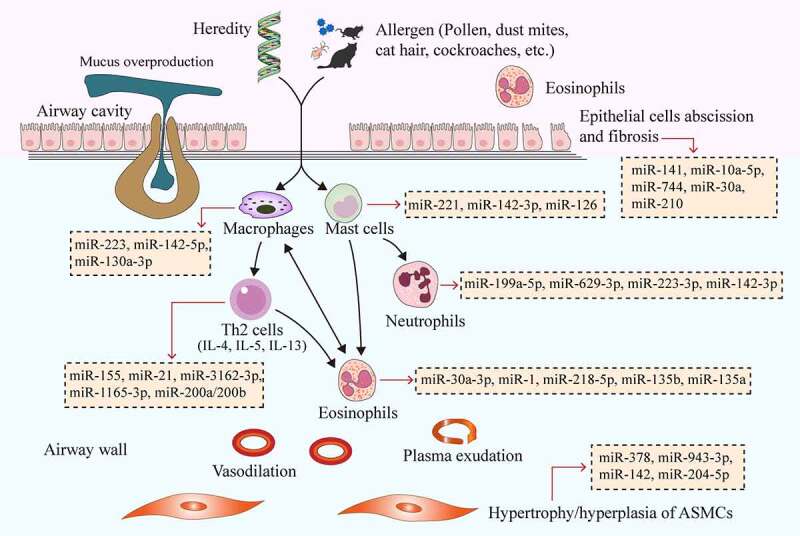
MiRNAs affect the pathogenesis of asthma by regulating immune cells, bronchial epithelial cells and ASMCs. Genetic and environmental factors (e.g., pollen, dust mites, cat hair, cockroaches and other allergens) cause the interaction of airway inflammatory cells (Th2 cells, eosinophils, mast cells, neutrophils, and macrophages), cytokines (e.g., IL-4, IL-5, and IL-13) and inflammatory mediators, ultimately leading to increased airway eosinophilia, mucus overproduction, airway remodeling, airway wall vasodilation, plasma exudation and airway epithelial cell exfoliation and fibrosis. Different miRNAs have different effects on the above processes. The yellow boxes in the picture show typical miRNAs that function in asthma, which correspond to the cells they act on through red arrows. MiRNAs, microRNAs; ASMCs, airway smooth muscle cells

Asthma is one of the most intractable chronic airway inflammatory diseases, which is driven by both genetic factors and environmental stimuli. Main symptoms include repeated wheezing, chest tightness, cough and other symptoms caused by reversible airflow restriction. More than 300 million people around the world are suffering from asthma [1], with an incidence of 7% to 10%. The pathogenesis of asthma involves genetic, immune, environmental allergens and pathogens. The co-participation of various immune cells and cytokines resulted in highly heterogeneous ranging from pathologic process to clinical manifestations. T helper 2 cells (Th2 cells) and type 2 cytokines (e.g., IL-4, -5 and -13) promote airway eosinophilia, bronchial hyperresponsiveness, mucus overproduction, airway remodeling and immunogloubulin E (IgE) synthesis in asthma [2] (Figure 1). Currently, treatments for asthma mainly include the avoidance of allergens and irritants as well as the use of beta-adrenergic receptor agonists, glucocorticoids, leukotriene modulators or immunotherapy. These methods can only alleviate the symptoms of asthma patients and keep the disease under control, but are not able to inhibit the recurrence of asthma or cure them completely [3]. There are still potential molecular mechanisms involved in the occurrence and development of asthma, and we need to further elucidate them in order to better treat asthma. Recently, the effect of non-coding RNAs (ncRNAs) on asthma has attracted researchers’ attention as a new mechanism, which may contribute to a better treatment of asthma.

**Figure 2.**
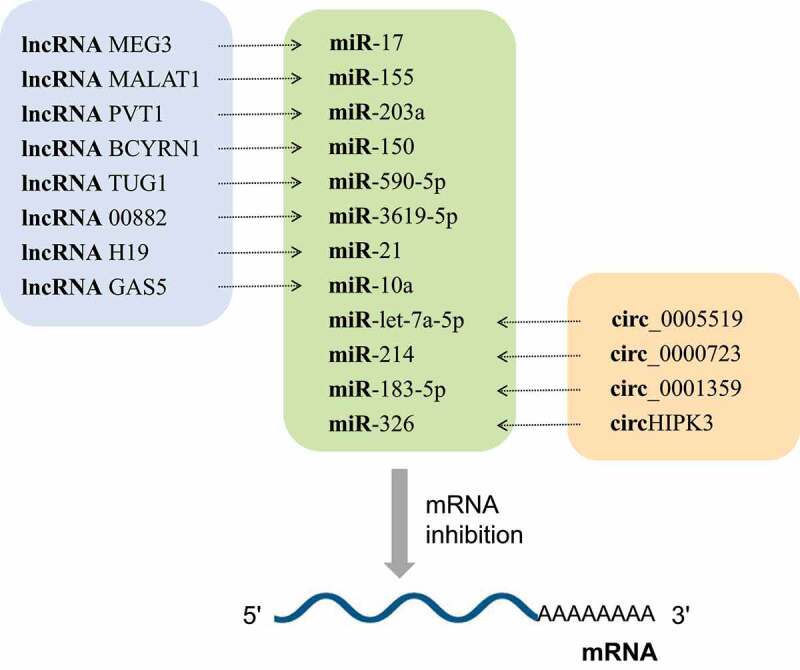
Most IncRNAs and circRNAs are involved in the pathogenesis of asthma through the lncRNA-miRNA-mRNA axis and circRNA-miRNA-mRNA axis, respectively. MiRNAs act as hub nodes in these axis. Different lncRNAs and circRNAs regulate different miRNAs through the sponge effect, and then inhibit mRNA

**Figure 3.**
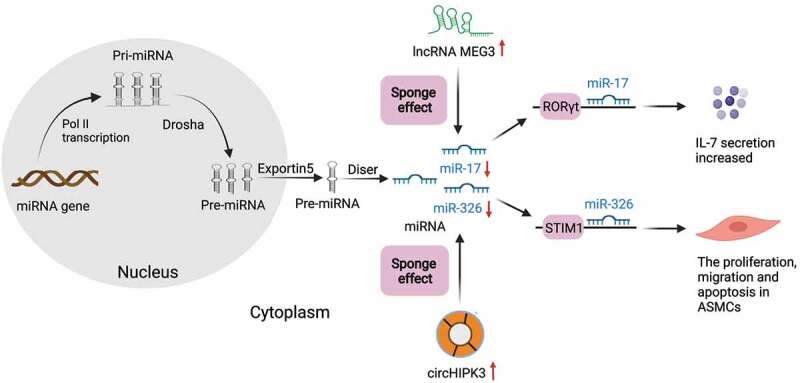
Schematic expression of regulation by ceRNAs during asthma. Pre-miRNA is generated in the nucleus and transfer to cytoplasm via exportin5. LncRNAs (green) and circRNAs (Orange circle) can influence miRNAs (blue) through the sponge effect in the cytoplasm in asthma. As the picture shows, the overexpression of lncRNA-MEG3 could competitively sponge miR-17 in asthma to regulate the expression of RORγt and ultimately affect the balance of Treg/Th17. Overexpressed circHIPK3 could sponge miR-326 in the cytoplasm, and then promote proliferation, migration and down-regulate the apoptosis in ASMCs by acting on STIM1

NcRNAs are a group of non-protein-coding RNA molecules that widely found in eukaryotes, and have been extensively studied in human diseases [4–7]. With the further development of molecular detection technology, more and more researchers have studied that ncRNAs play a key role in the occurrence and progression of asthma by regulating gene transcription [8]. This mainly includes the roles of circular RNAs (circRNAs), long non-coding RNAs (lncRNAs) and microRNAs (miRNAs) in asthma. MiRNAs are involved in the regulation of gene expression at the post-transcriptional level by targeting their corresponding mRNAs, and their regulatory roles in asthma are shown in Table 1. The effect of lncRNAs on asthma is mainly achieved by interfering with the expression of downstream genes, supplementing or interfering with the mRNA splicing process, and regulating the protein activity [9]. Besides, a growing body of research has found that lncRNAs can regulate gene transcription through the function of competing endogenous RNAs (ceRNAs; Table 2). As a new gene regulatory mechanism, ceRNAs have become more prominent recently, whereby some lncRNAs and circRNAs share the same miRNA response element (MRE). On this basis, they can indirectly inhibit the expression of miRNA to weaken the miRNA–mRNA interaction, and ultimately affect the function of cells by regulating gene expression levels. This action is termed as ‘sponging’ [10] (Table 2; Table 3; Figure 2; Figure 3). As a new transcriptional regulation model, the ceRNA networks can be employed to reveal how the entire network affects post-transcriptional regulation. CircRNAs are another type of ncRNAs with a unique covalent ring structure, which is formed during the abnormal splicing of protein-coding transcripts. Despite their low expression, some circRNAs have been found to influence the development of asthma by interfering with the maturation of legitimate protein-coding transcripts through ceRNA regulatory networks (Table 3).

**Table 1.** The role of miRNAs in the pathogenesis of asthma

MiRNA	Unusual expression	Targets	Function	References
Immune cells				
Th2 cells				
miR-155	Up	CTLA-4	Enhances the secretion of IL-4, -5, -13, -17a; enhances mucus secretion; stimulates T-cell activation and proliferative response	[20,27]
miR-1	Down	-	Inhibits the secretion of IL-4, -5, -8, TNF-α; regulates Th1/Th2 balance	[25]
miR-21	Up	IL-12p35 3ʹUTR	Modulates Th1/Th2 polarization	[31]
miR-3162-3p	Up	β-catenin	Enhances bronchial hyperresponsiveness and airway inflammation; regulates Th1/Th2 balance	[34]
miR-1165-3p	Up	IL-13,PPM1A	Inhibits bronchial hyperresponsiveness, airway inflammation, and differentiation of T cells toward Th2	[35]
miR-200a /200b	Down	ORMDL3	Inhibits the secretion of TNF-α, IL-4, -5, -13, and -1β	[36]
Eosinophils				
miR-30a-3p	Down	CCR3	Inhibits the secretion of specific IgE, eotaxin-1, IL-5, and IL-4	[39]
miR-218-5p	Down	CTNND2	Inhibits bronchial hyperresponsiveness, eosinophilic airway inflammation, and the expression of CCL26	[41]
miR-135b	Down	CXCL12	Inhibits the immune response of Th17 cells, goblet cell proliferation, and bronchial hyperresponsiveness; reduces the number of eosinophils and lymphocytes	[42]
miR-135a	Down	The STAT family	Inhibits bronchial hyperresponsiveness and lung pathological changes; reduces the secretion of TNF-α, IL-6, IL-5, and Eotaxin	[43]
Mast cells				
miR-221	Up	P27KIP1; PTEN and the p38/NF-κB pathway	Reduces the permeability of inflammatory cells, regulates the cell cycle of mast cells, and promotes their proliferation; increases the secretion of IL-4 and promotes the differentiation of Th cells into Th2 cells	[44–46]
miRNA-142-3p	Up	LPP	Enhances FceRI-mediated degranulation and rescues the reduction of degranulation	[47]
miRNA-126	Up	DNMT1	Increases the number of mast cells	[48]
Neutrophils				
miR-629-3p	Up	IL-8 mRNA	Increases the levels of IL-1β and IL-8 protein, which is positively correlated with the increase of neutrophils in sputum	[49]
miR-223	Up	NLRP3	Inhibits the airway inflammation, NLRP3 level and IL-1β release	[51]
Macrophages				
miR-142-5p	Up	SOCS1	Modulates the M2 polarization and profibrotic activities	[55]
miR-130a-3p	Down	Proliferator-activated receptor γ	Modulates the M2 polarization and profibrotic activities	[55]
Bronchial smooth muscle cells				
miR-26a	Up	GSK-3β	Promotes ASMCs hypertrophy	[57]
miR-378	Up	The genes related to ErbB, RAS, MAPK and calcium signaling pathway	Promotes ASMCs proliferation and apoptosis resistance	[59]
miR-19a	Up	PTEN	Inhibits ASMCs proliferation and migration	[60]
miR-943-3p	Up	SFRP4	Increases the number of macrophages, eosinophils, lymphocytes, and neutrophils, increases the expression of collagen, β-catenin, and c-Myc in lung tissue, and promotes subepithelial fibrosis and ASMCs proliferation	[61]
miR-142	Down	The genes related to EGFR signaling pathway	Inhibits the proliferation of ASMCs and promotes their apoptosis	[62]
miR-204-5p	Down	SIX1	Inhibits ASMCs proliferation and extracellular matrix production	[63]
Bronchial epithelial cells				
miR-744	Down	TGF-β1	Inhibits bronchial epithelial cell proliferation	[119]
miR-141	Up	CLCA1	Stimulates IL-13-induced goblet cell metaplasia, promotes airway remodeling and pathological airway mucus production	[67]
miR-146a	Up	IRAK1, TRAF6, FOXO3, PDE7A	Promotes pulmonary mucin deposition, airway remodeling, IgE synthesis, and eosinophilic inflammation	[68,69]
miR-210	Down	FOXP3	Involves in the polarization of Th2 cells	[72]
miR-34a	Down	The genes related to Wnt signaling pathway	Involves in the polarization of Th2 cells	[72]

**Table 2.** Function of lncRNAs involved in the pathogenesis of asthma

Name of lncRNA	MiRNA sponge	Species/ Cells	Targets	Functions	References
lncRNA MEG3	miR-17	Human/ CD4^+^ T cell	RORγt	IL-17 secretion; Treg/ Th17 balance	[78]
lncRNA MALAT1	miR-155	Human/CD4^+^ T cell	T-bet, GATA-3	Th1/ Th2 balance	[79]
lncRNA RMRP	miR-155	Mice/ CD4^+^ T cell	CCL2	Th1/ Th2 balance	[94]
lncRNA PVT1	miR-203a	Rat/ ASMC	E2F3	The proliferation and migration of ASMCs	[89]
lncRNA PTPRE-AS1	-	Human, mice/Macrophage	PTPRE	M2 macrophage activation	[81]
lnc-BAZ2B	-	Human, mice/Macrophage	BAZ2B	M2 macrophage activation	[82]
lncRNA-AK149641	-	Mice/Mast cell	NF-κB	The inflammatory response in mast cells	[83]
lncTCF7	-	Human/ ASMC	TIMMDC1	The proliferation and migration of ASMCs	[87]
lncRNA MALAT1	miR-150	Human/ ASMC	eIF4E	The proliferation and migration of ASMCs	[90]
lncRNA BCYRN1	miR-150	Rat/ ASMC	-	The proliferation and migration of ASMCs	[120]
lncRNA TUG1	miR-590-5p	Rat/ ASMC	FGF1	The proliferation and migration of ASMCs	[91]
lncRNA TUG1	miR-181b	Rat/ ASMC	HMGB1	The proliferation and migration of ASMCs	[95]
lncRNA 00882	miR-3619-5p	Human/ fetal ASMC	β-catenin	The proliferation of ASMCs	[92]
lncRNA H19	miR-21	Human/ ASMC	PTEN	The proliferation and migration of ASMCs	[96]
lncRNA CASC7	miR-21	Human/ ASMC	PTEN	Regulating PI3K/ AKT signaling pathway; corticosteroid resistance	[99]

**Table 3.** Function of circRNAs involved in the pathogenesis of asthma

Name of circRNA	MiRNA sponge	Species/ Cells	Targets	Functions	References
circ_0005519	Let-7a-5p	Human/ CD4^+^ T cell	IL-13, IL-6	IL-13 and IL-6 expression	[107]
circ_0000723	miR-214	Mice/ CD4^+^ T cell	RUNX	Th1/ Th2 balance	[115]
circ_0001359	miR-183-5p	Mice/ macrophage	FoxO1	M2 macrophage activation	[116]
circHIPK3	miR-326	Human/ ASMC	STIM1	The proliferation, migration and apoptosis in ASMCs	[117]

In this paper, we focused on the regulatory functions and molecular mechanisms of ncRNAs (circRNAs, lncRNAs, and miRNAs) and ceRNA regulatory networks in the pathogenesis of asthma, which could provide novel ideas for better understanding the disease pathogenesis and identifying novel diagnostic biomarkers. This could also offer a theoretical basis for the development of new anti-asthma therapies based on the regulatory potential of ncRNAs.

## 2. MiRNAs in asthma

MiRNAs are small ncRNAs composing of about 19–25 nucleotides, which are highly conservative in the process of biological evolution. MiRNAs can bind to mRNA in a 3ʹ-untranslated region, which in turn degrades mRNA and inhibits translation, thereby modifying other cellular functions [11]. At present, we have found that miRNAs play essential roles in regulating cell proliferation and differentiation, signal transduction, stress response, cell apoptosis, and other aspects. Differentially expressed miRNAs were detected in the pulmonary tissue of mouse asthma model, which could regulate transforming growth factor-β (TGF-β), matrix metalloproteinases and other inflammatory and apoptotic signaling pathways [12], thus playing vital roles in the occurrence and progression of asthma. MiRNAs can affect Th1/Th2 polarization, airway remodeling, and chronic epithelial inflammation by acting on bronchial epithelial cells, airway smooth muscle cells (ASMCs), and immune cells [13] (Figure 1), which provide a new idea for the molecular diagnosis and treatment of asthma [14]. In addition, miRNAs may also serve as a new biomarker for the diagnosis, phenotypic determination, condition assessment or prediction of the future course of this disease [11].

### 2.1 The role of miRNAs in immune cells

As important regulatory factors of the immune system, miRNAs play an important role in mediating inflammatory effector cells (e.g., Th cells, eosinophils, mast cells, neutrophils, and macrophages). In the vast majority of cases, asthma is caused by the T2-high phenotype. Previous studies have suggested that the role of most miRNAs in asthma is associated with eosinophilia (Th2 type) and airway inflammation (T2-high asthma). MiRNAs are involved in asthma-related inflammatory processes by regulating the activation and differentiation of Th2 cells, secretion of cytokines, and functions of eosinophils [15–19]. There are also potential relationships between miRNAs and mast cells, neutrophils and macrophages, but their exact role in asthma needs to be further evaluated. The regulation of miRNA to immune cells is shown in Figure 1.

#### 2.1.1 MiRNAs in Th2 cells

Recently, several studies have explored the role of miRNAs in asthma-related inflammatory processes through regulating T cell function. The findings showed that miRNAs participate in asthma-related inflammatory processes by modulating the activation and differentiation of Th cells as well as the secretion of cytokines (Table 1). The regulatory effect of miRNAs on Th2 cells and their cytokines is more extensive. It has been reported that miR-1, -18a, -21, -146a, -155, -210 and -1248 can regulate the function of T cells and the production of Th2 cytokines, which are involved in the pathogenesis of asthma [20–26]. MiR-155 can enhance the proliferation of T cells by suppressing cytotoxic T lymphocyte-associated protein 4 (CTLA-4). The absence of miR-155 can promote the differentiation of T cells into Th2 by increasing IL-4 secretion, but prevent the activation of Th2 triggered by dendritic cells *in vivo*, which may be helpful for the investigation of asthma treatment [20,27]. Recent studies demonstrated that the blood level of miR-1 was lower in children with acute asthma attack than in healthy children, and had a negative correlation with the expression of tumor necrosis factor-alpha (TNF-α), IL-4, -5 and -8 [25]. However, as the severity of the disease increased, its correlation became stronger [25]. Thus, miR-1 can be used to diagnose the deterioration of asthma condition and evaluate the severity of the disease [25]. MiR-21 was significantly increased in both house dust mite- and ovalbumin (OVA)-induced mouse lungs [28,29], and it was also involved in the inflammatory mechanism of asthma by inhibiting Th1 differentiation and thereby enhancing Th2 polarization [30,31]. Inhibition of miR-21 could downregulate CD4^+^/CD8^−^ T cell ratio and Th2 cytokine levels in the spleen of asthmatic model mice by targeting IL-12p35 [31,32]. However, in another study [33], miR-21 antagonists did not significantly affect Th2 cytokine production after intranasal sensitization in mice. Accordingly, miR-21 may act in the early sensitization stage and it is promising to become a therapeutic target for early asthma.

Further research has shown that the blood level of miR-3162-3p was significantly higher in children with asthma than in healthy children [34]. Inhibition of miR-3162-3p in OVA-induced asthmatic mice could effectively alleviate airway inflammation and bronchial hyperresponsiveness, decrease β-catenin expression, maintain Th1/Th2 balance and inhibit airway remodeling [34]. When allergic airway inflammation occurs, miR-1165-3p plays an effective role by targeting IL-13 and protein phosphatase, Mg2^+^/Mn2^+^-dependent 1A (PPM1A). It inhibits bronchial hyperresponsiveness, airway inflammation, and differentiation of T cells toward Th2 in patients with asthma through negative regulation of IL-13 and PPM1A [35]. Upregulated miR-200a/200b expression could inhibit the ERK/MMP-9 pathway by targeting orosomucoid 1-like 3 (ORMDL3), reduce the secretion of TNF-α, IL-1β, -4, -5 and -13, and slow down the development of asthma-related inflammation [36]. Hence, the increased expression of miR-200a/200b may be potential therapeutic targets for asthma. Collectively, miRNAs can regulate the pathogenesis of allergic airway inflammation in asthma by affecting the role of Th2 cells.

#### 2.1.2 MiRNAs in eosinophils

Eosinophils are important inflammatory cells involved in the pathogenesis of asthma (Figure 1). It has been noted that eosinophils produce a large number of cytokines during eosinophil activation, which exert different roles in the immune responses of Th1 and Th2 [37]. It has been reported that miR-181a, -146a and -146b were positively correlated with total cell number or eosinophil count in the bronchoalveolar lavage fluid (BALF) of OVA-induced murine asthma model [38]. MiR-30a-3p could negatively regulate the CC chemokine receptor (CCR3) signaling pathway, reduce the secretion of specific IgE, eosinophil chemokine-1 (eotaxin-1), IL-5 and IL-4 in OVA-induced asthmatic mice, and suppress the asthmatic inflammatory responses [39]. Korde et al. [40] proposed for the first time that endothelial miR-1 could regulate eosinophil transport. The high level of endothelial miR-1 inhibited airway eosinophil proliferation and allergic airway inflammation by regulating P-selectin (SELP), eotaxin-3 (CCL26), thymic stromal lymphopoietin (TSLP), and thrombopoietin receptor (MPL) [40]. Overexpression of epithelial miR-218-5p could relieve eosinophilic airway inflammation, inhibit bronchial hyperresponsiveness and decrease chemokine CCL26 expression by targeting CTNND2 (encoding δ-catenin) [41]. In addition, miR-135b could inhibit the immune response of Th17 cells, goblet cell proliferation, and bronchial hyperresponsiveness as well as reduce the number of eosinophils and lymphocytes in OVA-induced asthmatic mice through negative regulation of CXCL12 [42]. MiR-135a agonists could improve bronchial hyperresponsiveness, attenuate lung pathological changes, and reduce the expression of eotaxin, TNF-α, IL-5 and IL-6 in the BALF and lung tissue of asthmatic mice via activating JAK/STAT signal transduction [43] (Table 1). Therefore, miRNAs play a crucial role in the regulation of eosinophils in asthma, which needs to be further studied.

#### 2.1.3 MiRNAs in mast cells

Mast cells are the key effector cells involved in IgE-related allergic diseases, and have an immunomodulatory function. When exposed to allergens, mast cells can release inflammatory regulatory factors, which are also regulated by miRNAs during their formation (Table 1). In recent years, few studies reported that miR-221 inhibition could reduce the permeability of inflammatory cells by targeting P27KIP1, regulate the cell cycle in mast cells, and promote cell proliferation [44,45]. Through the study of asthmatic children and an OVA-induced murine asthma model, Zhou et al. [46] found that miR-221 overexpression could stimulate mast cells to secrete IL-4 by targeting PTEN, p38, and nuclear factor‑kappa B (NF-κB) signaling pathways, and subsequently promote the transformation of Th cells into Th2 cells. Yamada et al. [47] also found that miR-142-3p overexpression could enhance FcεRI-mediated mast cell degranulation and rescue the reduced degranulation by silencing Dicer. Therefore, miR-142-3p may be used as a target for allergic reaction therapy. A recent study has also shown that miR-126 may act on the target gene DNMT1 and eventually affect the number of mast cells in asthma [48]. Although numerous miRNAs are related to the activation, proliferation and differentiation of mast cells, the functional role of miRNA in mast cells still needs to be further elucidated.

#### 2.1.4 MiRNAs in neutrophils

Neutrophilic inflammation-related asthma (T2-low asthma) is currently uncommon and does not respond well to conventional asthma treatment. The lack of biomarkers to identify this phenotypic disease makes it a major challenge for asthma treatment. The expression levels of miR-142-3p, -199a-5p, -223-3p and -629-3p in the sputum of patients with neutrophilic asthma were significantly increased compared to those in healthy subjects. These miRNAs were involved in the inflammatory process of asthma. MiR-142-3p and -223-3p were overexpressed in macrophages, monocytes, and neutrophils; while miR-629-3p was overexpressed in bronchial epithelium [49]. The expression of miR-199a-5p was negatively correlated with pulmonary function damage in patients with neutrophilic asthma, which plays a major role in the pathogenesis of the disease by modulating inflammatory process [50]. The levels of IL-1β, IL-8 and neutrophils were significantly elevated in the sputum of patients with severe asthma, and there was a positive correlation among them [49]. MiR-629-3p could induce the mRNA and protein expression of IL-8 in human bronchial epithelial cells. Xu et al. [51] found that miR-223 could regulate airway inflammation, NLRP3 level, and IL-1β release by negatively regulating NLRP3/IL-1β axis, thus contributing to immune responses during neutrophilic asthma. Besides, neutrophil-derived miR-223-3p in the sputum of asthma patients could regulate Toll-like receptors (TLRs)/Th17 signal transduction and endoplasmic reticulum stress response [52] (Table 1). However, the actual relationship between miRNAs and neutrophilic inflammation-associated asthma needs to be further investigated.

#### 2.1.5 MiRNAs in macrophages

M1 and M2 are the two main polarization states of macrophages. M1 cells are a classical type of pro-inflammatory activation cells induced by interferon gamma (IFN-g), TNF and TLR; while M2 cells are alternately activated for anti-inflammation induced by IL-4, -10 and -13. The imbalance between M1 and M2 cells promotes the occurrence of asthma. The role of miRNAs in regulating macrophages in eosinophil and neutrophilic asthma has recently been investigated. MiR-9, -27, -125b and -155 can induce the M1 phenotypic transformation of macrophages; whereas miR-21, -34, -146a, -223 and let-7c can provoke anti-inflammatory response and the M2 phenotypic transformation of macrophages [53]. However, their exact roles in the pathogenesis of asthma have not been fully understood. Previous research demonstrated that miR-142-5p and miR-130a-3p can regulate the expression of macrophage fibrogenic genes, which ultimately affect tissue fibrosis. The specific mechanisms are as follows. STAT6 is a key transcription factor that regulates the polarization of M2 macrophages. The upregulation of miR-142-5p is related to the inhibition of SOCS1 expression, leading to prolong phosphorylation time of STAT6. Reduced expression of miR-130a-3p alleviated the inhibition of proliferator-activated receptor γ expression, thereby promoting the STAT6 response [54]. Recent studies have found that Th2 cytokines can upregulate miR-142-5p and downregulate miR-130a-3p in an OVA-based murine model of asthma, thus affecting the M2 polarization and profibrotic activities of pulmonary macrophages. These results suggest that miR-142-5p and -130a-3p can act as regulators of pulmonary macrophage polarization and related airway remodeling in asthma [55] (Table 1). Therefore, miRNA may influence the severity of asthma by regulating the role of macrophages. However, the detailed molecular mechanism still needs to be further explored.

### 2.2 MiRNAs in airway smooth muscle cells

Hypertrophy and proliferation of ASMCs are signs of bronchial remodeling in asthma, and ASMCs are also the main cell type associated with the pathophysiological processes of asthma [56]. Increasing evidence has shown that miRNA may regulate the excessive proliferation, enhanced migration and decreased apoptosis of ASMCs (Table 1). Mohamed et al. [57] revealed for the first time that miR-26a is a mechanically sensitive gene and enforced expression of miR-26a could induce vascular smooth muscle cell hypertrophy via targeting glycogen synthase kinase-3β (GSK-3β) pathway. The important regulators of ASMCs, miR-25 and -145, are involved in the pathogenesis of asthma by targeting Krüppel-like factor 4 (KLF4). KLF4 can be used as a specific inhibitor of ASMCs gene expression and an inflammatory mediator that participates in airway remodeling [58]. Related research showed that miR-378 expression is increased in the lung tissue and blood samples of asthma children compared to normal controls. MiR-378 targets the genes related to ErbB, MAPK, RAS and calcium signaling pathways, thereby enhancing the proliferation and reduces the apoptosis of ASMCs [59]. Downregulated expression of miR-378 can inhibit the proliferation of ASMCs by affecting the cell cycle. MiR-19a may contribute to the inhibition of ASMCs growth and migration by targeting PTEN3ʹ-UTR and regulating HMGB1, and the miR-19a/PTEN/AKT axis is a key signaling pathway that mediates the inflammatory responses of ASMCs [60]. MiR-943-3p has been shown to suppress secreted frizzled-related protein 4 (SFRP4) activity in asthma and OVA-induced asthmatic mice through regulating Wingless/Integrase-1 (Wnt) signaling pathway. This leads to the increased numbers of macrophages, eosinophils, lymphocytes, neutrophils and other inflammatory cells, the upregulated expression of collagen, β-catenin and c-Myc in lung tissues, subepithelial fibrosis and smooth muscle thickening, thus aggravating the processes of airway remodeling and asthmatic inflammation [61]. Collectively, miR-19a, -25, -378, -26a, -145 and -943-3p are all miRNAs that can promote airway remodeling. However, some miRNAs have been shown to inhibit the proliferative effects of ASMCs during asthma. For example, miR-142 inhibits the expression of TGF-β by regulating epidermal growth factor receptor (EGFR) signaling pathway, which in turn suppresses ASMCs proliferation and promote ASMCs apoptosis in asthmatic rats during airway remodeling [62]. MiR-204-5p may become a potential therapeutic target for the prevention of airway remodeling in asthma. The overexpression of miR-204-5p inhibits TGF-β1-induced proliferation of ASMCs and production of extracellular matrix proteins by regulating SIX1 [63]. Therefore, new miRNAs-targeted ASMCs remodeling strategies may have important implications for the treatment of asthma patients.

### 2.3 Functional role of miRNAs in epithelium

Epithelial cells can be used to resist the external environment and secrete mucus for capturing inhaled particles and pathogens, thus playing an essential role as a physical barrier [64,65]. Bronchial epithelium is a key factor for the control of airway wall remodeling, which can participate in the pathogenesis of asthma by secreting cytokines and regulating inflammatory process. MiRNAs can influence asthma through their activity in epithelial cells [66] (Table 1). Recently, Siddiqui et al. [67] have found that miR-141 antagonists can reduce bronchial hyperresponsiveness and mucus production without causing inflammation. Thus, when T2-high asthma occurs, miR-141 inhibition can play significant roles in attenuating mucus production and airflow obstruction, which has become one of the new strategies for the treatment of asthma. Studies have shown that miR-10a-5p and -146a-5p are upregulated in the epithelial cells of patients with asthma and COPD, and they may play vital roles in regulating biological processes (e.g., apoptosis and inflammation) and immune cell activities (e.g., eosinophils, neutrophils, and T cells) by targeting FoxO3 and PDE7a [68,69]. On the contrary, Pacholewska et al. have shown that miR-744 is remarkably downregulated in patients with severe asthma, and its overexpression may inhibit the proliferation of bronchial epithelial cells and regulate Smad3 signaling pathway via targeting TGF-β1 [70]. MiRNAs can affect bronchial fibrosis by regulating epithelial cells. Li and colleagues demonstrated that miR-30a expression was downregulated in childhood asthma and OVA-induced asthmatic mice, while the expression of autophagy-related proteins was upregulated. MiR-30a overexpression alleviated IL-33-induced bronchial epithelial fibrosis and inhibits autophagy by downregulating autophagy-associated protein 5 (ATG5). Therefore, miR-30a has anti-fibrotic effects on IL-33-induced lung epithelial cells *in vitro* and on OVA-induced mouse airway inflammation model *in vivo*, which may be used in the future treatment of asthma [71].

The extracellular miRNAs secreted by human bronchial epithelial cells can also influence the occurrence and development of asthma. Wiley et al. [72] investigated the miRNA changes in extracellular vesicles produced from IL-13-induced primary normal human bronchial epithelial (NHBE) cells during the development of asthma, and the findings indicated that miR-34a, -92b and -210 were related to pulmonary functions. Moreover, the secretion of miRNAs (e.g., miR-34a, -92b, and -210) in extracellular vesicles from the airway epithelium might be involved in the polarization of Th2 cells in asthmatic airway. These studies suggest that the abnormal expression of miRNAs is closely related to the enhanced pro-inflammatory pathway activity, altered expression of tight junction proteins, or other observed epithelial changes in asthma patients.

## 3. LncRNAs in asthma

LncRNAs are linear RNAs that are more than 200 nucleotides long, do not encode proteins and widely occur in various cells. LncRNAs play an important role in gene regulation, including recruiting chromatin modifiers, blinding to transcription factors, formatting ribonucleoprotein complexes, and inhibiting translation, etc [73,74]. Although only a small number of studies have proved that lncRNAs can directly mediate the pathogenesis of asthma, many studies have been that lncRNAs and asthma are inextricably linked. Researchers recently found that lncRNAs can exert their biological effects through ceRNA regulatory network to competitively combine with miRNAs [75]. Further studies have shown that most lncRNAs can affect the occurrence and development of diseases by acting as ceRNAs (Table 2; Figure 2). It has been found that 31 genes, including three lncRNAs of long intergenic non-protein coding RNA 1959, KIAA0087 LncRNA, and long intergenic non-protein coding RNA 2209, can be used as the optimal asthma biomarker [76]. The study of the expression of lncRNA in asthma is helpful to further determine the biomarkers and therapeutic targets of asthma.

In recent years, increasing numbers of studies have suggested that lncRNAs participate in the occurrence and progression of bronchial asthma by affecting immune response, inflammatory response and cytokine expression [77]. LncRNAs are associated with immune regulation by regulating the development of T cells. A recent study showed that lncRNA-MEG3 could competitively sponge miR-17 in the CD4^+^ T cells of asthma patients to regulate the expression of RORγt and ultimately affect the balance of Treg/Th17 [78] (Figure 3). Liang et al. [79] found for the first time that lncRNA MALAT1 sponging miR-155 could negatively regulate miR-155 expression and subsequently alter the Th1/Th2 balance within CD4^+^ T cells through a CTLA-4-dependent mechanism. However, whether targeting the MALAT1/miR-155/CTLA-4 axis can change Th2-dependent response that promotes airway inflammation in asthma is not completely clear and needs further study. Upregulated lncRNA PVT1 could inhibit the expression of miR-149 in bronchial epithelial cells, promote airway inflammation and destroy the cellular barrier, thus accelerating the development of asthma [80]. It is speculated that PVT1 may be a new potential target for the treatment of asthma. In recent years, much attention has been paid to the effect of lncRNAs on the pathogenesis of asthma mediated by macrophages. It has been found that lncRNA PTPRE-AS1 targets receptor-type tyrosine-protein phosphatase ε (PTPRE) and ultimately regulates the activation of M2 macrophages. When PTPRE-AS1 is deficient, it can enhance the activation of IL-4-mediated M2-like macrophages, thus exacerbating pulmonary allergic inflammation [81]. Lnc-BAZ2B induces M2-like macrophage activation by increasing the expression of BAZ2B and aggravates pulmonary inflammation in an M2-like macrophage-related cockroach allergen extract-induced mouse models. Thus, it can be used as a potential target for the treatment and diagnosis of childhood asthma [82]. Recently, Yao et al. [83] have focused on the effect of lncRNA-AK149641 on NF-κB signaling pathway, and the results showed that this lncRNA could bind to NF-κB in the nucleus. Low NF-κB DNA nuclear binding was observed in mast cells with downregulated lncRNA-AK149641 expression, and lncRNA-AK149641 could regulate lipopolysaccharide (LPS)-induced mast cell inflammation by regulating NF-κB signaling pathway. Therefore, it is speculated that this mechanism has a similar effect on asthma. Li and coworkers found that lncRNA NEAT1 expression was negatively correlated with miR-124 in asthma patients [84]. Besides, miR-124 was negatively correlated with the high risk, severity and inflammation of asthma, but positively correlated with lung function. It has also been demonstrated that lncRNA NEAT1 may be involved in the aggravation of asthma by sponging miR-124. Similarly, lncRNA ANRIL/miR-125a has been found to be a possible predictor of disease deterioration, severity, and inflammation in bronchial asthma [85]. These results suggest that lncRNAs are associated with the occurrence and progression of asthma, which potentially serve as new targets for treating asthma.

The proliferation and migration of ASMCs can lead to airway wall thickening, which is involved in airway remodeling and irreversible bronchoconstriction in asthma [86]. The regulatory roles of lncRNA in ASMCs have been increasingly reported over recent years, especially through the mechanism of lncRNA-miRNA-mRNA regulatory network. Fan et al. found that lncTCF7 is highly expressed in asthma group, activates the signal transduction of AKT, and ultimately promotes the growth and migration of ASMCs by targeting TIMMDC1 [87]. Several studies have shown that lncRNA growth arrested-specific 5 (lncRNA GAS5), lncRNA PVT1, lncRNA MALAT1, lncRNA TUG1 and LINC00882 can act as the ‘sponge’ function of miR-10a, -150, -203a, -590-5p and -3619-5p, respectively, and then involved in the biological processes of ASMCs proliferation and migration in asthmatic patients [88–92]. Recently, Zhumx and colleagues demonstrated that the expression of lncRNA NEAT1 was increased and that of miR-139 was decreased in the ASMCs of asthma patients. LncRNA NEAT1 overexpression can activate JAK3/STAT5 signaling pathway by targeting miR-139, thus promoting the levels of inflammatory cytokines (e.g., TNF-α, IL-1β, -6 and -8) in ASMCs. From this, it can be inferred that lncRNA NEAT1 plays an important role in ASMCs inflammation through regulating miR-139/JAK3/STAT5 signaling axis, and may become a target for asthma therapy [93]. Yin et al. [94] found that RNA component of mitochondrial RNA-processing endoribonuclease (RMRP) upregulates the expression of C–C motif ligand 2 (CCL2) via competitively sponging miR-206. Overexpressed RMRP in ASMCs can inhibit the TGF-β/Smad2 signaling pathway by acting on the RMRP/miR-206/CCL2 axis, suppress inflammatory cytokines, enhance cell viability, promote cell apoptosis, and ultimately affect the occurrence and progression of asthma. Huang et al. [95] found that lncRNA TUG1 sequester miRNA-181b upregulates HMGB1 via NF-κB signaling pathway and promotes airway remodeling in asthmatic mice. Recent research has indicated that lncRNA KCNQ1OT1 plays a crucial role in airway remodeling of bronchial asthma, and its expression is significantly correlated with the thickness of reticular basement membrane and the quantity of fibroblasts in bronchial mucosa [96]. The detection of serum lncRNA KCNQ1OT1 can be used to monitor the occurrence and progression of airway remodeling in childhood asthma, thus providing a new idea and diagnostic tool for asthma therapy. LncRNA H19 overexpression reduces the growth and migration of platelet-derived growth factor-BB(PDGF-BB)-induced ASMCs by acting on miR-21/PTEN/Akt pathway, which can be used as a potential biomarker and therapeutic target for ASMCs proliferation [97]. The above results suggest that lncRNAs not only directly act on ASMCs, but also indirectly control miRNA target gene expression to regulate the growth and migration of ASMCs by interacting with miRNA. The proliferation of ASMCs can aggravate airway inflammation and then trigger asthma.

Besides, Wang et al. [98] for the first time studied the association between lncRNA and induced pluripotent stem cell-derived mesenchymal stem cells (iPSC-MSCs) in allergic airway inflammation. They found that the expression levels of MM9LINCRNAEXON12105+ and AK089315 are increased in the asthma model and reduced after treatment with iPSC-MSCs, suggesting that lncRNA may be involved in allergy and immune regulation of iPSC-MSC. Liu et al. [99] found that lncRNA CASC7 inhibit PI3K/Akt signaling pathway through targeting miR-21 and promote the phosphorylation of glucocorticoid receptors to increase the sensitivity of glucocorticoids in patients with severe asthma. In a study involving the pathogenesis of cigarette smoke-induced COPD, lncRNA TUG1 promoted airway inflammation and airway remodeling by downregulating miR-145-5p/dual-specificity phosphatase 6 (DUSP6) axis in CS extract (CSE)‐treated human bronchial epithelial cells and lung fibroblasts [100]. Considering the important role of bronchial epithelial cells in Th2-type immune response and airway mucoid metaplasia, it is speculated that lncRNA TUG1 can also act as a ceRNA in bronchial epithelial cells of asthma, which needs to be further explored. In another study, Liao et al. [101] constructed an asthma-related ceRNA network using online databases (e.g., Gene Expression Omnibus, StarBase, DrugBank and bioinformatics tools), and they identified 5 key lncRNAs (i.e., CASC2, DAPK1-IT1, MAGI2-AS3, MALAT1, and MIR17HG) and predicted 8 therapeutic drugs (i.e., dasatinib, glyburide, levocarnitine, niflumic acid, quercetin, ruxolitinib, tamoxifen, and tretinoin) with corresponding ceRNA targets. Therefore, lncRNAs can regulate the occurrence of asthma by interacting with miRNAs and indirectly regulating miRNA target gene expression, and the lncRNA-targeting drugs deserve further exploration.

The above studies have shown that lncRNAs are closely related to the occurrence and development of asthma, but few studies have been conducted on the direct regulation of lncRNAs in asthma. Most IncRNAs are involved in the pathogenesis of asthma through the lncRNA-miRNA-mRNA axis, which plays a vital role in multiple diseases such as endometrial carcinoma, ovarian cancer and osteosarcoma [102–104]. The analysis on the role of ceRNA regulatory networks in other diseases also provides a new idea for further study of asthma. Since the expression profile of lncRNA and miRNA is different in different diseases, the lncRNA-miRNA-mRNA axis in asthma is specific to some extent in accordance with bioinformatic evidence, and it shows promise as a therapeutic target for asthma in the future, which needs to be confirmed further. Currently, only a small number of lncRNAs can be used as biological indicators for clinical judgment of asthma progression and prognosis evaluation of patients. With our further understanding of lncRNAs in asthma, the research of drugs related to lncRNAs may become a new direction in the field of targeted asthma therapy.

## 4. CircRNAs in asthma

CircRNAs are a special kind of ncRNAs that form a ring structure by a covalent bond and have no 5ʹ terminal cap and 3ʹ terminal poly (A) tail. CircRNAs can interact with proteins, and regulate gene splicing or transcription, protein or peptide translation, and epigenetically influence a variety of biological processes by competitively sponging miRNAs [105]. CircRNAs with miRNA binding sites can play a role as ceRNAs. CircRNA sequence is significantly enriched in conserved nucleotides and it can resist the degradation of exonuclease. Rich endogenous circRNAs can be used as effective miRNA ‘sponges’, thereby enriching the regulatory function of gene expression [106]. In recent years, circRNAs have attracted extensive attention in the pathogenesis of asthma (Table 3; Figure 2).

Huang et al. [107] analyzed the circRNA spectrum of CD4^+^ T cells in asthma patients, and the results showed that circ_0005519 could affect the secretion of IL-13/IL-6 through competitively sponging let-7a-5p to regulate T cell-mediated inflammatory process. This provides a new insight into the treatment of asthma. Recent studies have shown that circ_0002594 competitively suppresses miR-16-5p, -503-5p, -514a-3p, -587, and let-7e-5p, which may be valuable for the diagnosis and treatment of Th2-type asthma [108]. The regulatory networks of circRNAs and miRNAs showed that two up-regulated circRNAs (circ_0000629 and circ_0000455) could target miR-15a and miR-29b, respectively, which were negatively related to the development of allergic reaction [109,110]. MiR-29b can target inducible costimulatory factors (ICF) and promote the production of Th2 cytokines and eosinophilic inflammation [111]. MiR-15a is overexpressed in Th2-mediated pulmonary inflammation, such as asthma, induce asthma-like phenotype, and target vascular endothelial growth factor (VEGF) [112]. On the contrary, a ceRNA regulatory network, involving two downregulated circRNAs (circ_0000723 and circ_0001454) and their corresponding miR-214 and miR-146b, was positively correlated with the occurrence of hypersensitivity [38,113]. It has been reported that miR-146b further promoted or maintained the Th2-dependent response by inhibiting the activation of Th1 [114], while miR-214 could play a role in asthma by targeting Runx. The interaction between NFAT and Runx3 may cause the negative regulation of IL-4, which will eventually impact the balance of Th1/Th2 [115]. Thus, different circRNAs are expressed differently in asthma and have different effects on inflammatory cytokines in asthma. The mechanism of circRNAs on asthma should be further studied in order to seek for potential diagnostic and therapeutic targets of asthma. The roles of circRNAs in other immune cells have also been gradually discovered. For example, the exosomes from mmu_circ_0001359-modified adipose-derived stem cells (ADSCs) could sponge miR-183-5p and alleviated airway remodeling in M2-like macrophages activated by FoxO1 signal transduction [116]. CircRNAs also play a role in ASMCs. Lin et al. [117] found that overexpressed circHIPK3 could sponge miR-326 in the cytoplasm in asthma, and then promoted the proliferation and migration as well as inhibited the apoptosis of ASMCs by acting on stromal interaction molecule 1 (STIM1), which may become a new strategy for treating asthma (Figure 3). Recently, Bao et al. [118] found that circRNAs are also involved in the pathogenesis of allergic asthma through lipid metabolism, cell adhesion molecules and macrophage endocytosis. They proposed that circRNA expression might be positively associated with asthma, which provided a new idea for studying the etiology and pathogenesis of asthma. As a whole, the role of circRNAs in asthma is complex and needs to be further explored.

In conclusion, with further studies on the pathogenesis of asthma, the role of circRNAs in asthma has been gradually discovered. At present, there are more and more studies on the competitively sponging of circRNAs to miRNAs. Some studies found that circRNA-TBCD can competitively sponge miR-138 and circRNA-100242 can competitively sponge miR-145. However, the specific effect of these circRNAs in asthma is not completely clear. Whether these interactions can affect the occurrence and development of asthma still need to be further explored in the future research. CircRNAs may be used as a new diagnostic marker of asthma, and can be used as a new direction of targeted drug therapy. The specific mechanism warrants further experimental verification.

## 5. Conclusion and future direction

In summary, ncRNAs and ceRNA regulatory networks may provide new ideas for the diagnosis and treatment of asthma by regulating immune cells, ASMCs, and bronchial epithelial cells, which participate in the inflammatory response, airway remodeling, and bronchial hyperresponsiveness of asthma. However, the pathogenesis of asthma is not completely clear, the roles of ncRNAs and ceRNAs in the pathogenesis of asthma needs to be further studied in the near future. At present, it has been reported that there are few biomarkers and targeted drugs targeting ncRNAs using for clinical diagnosis and treatment, so it is necessary to verify the feasibility of ncRNAs in clinical asthma management. With the development of bioinformatics, basic immunology, RNA biology, genomics and proteomics, it will be more helpful to understand the pathogenesis of allergic asthma regulated by ncRNAs, thus making ncRNAs and ceRNA regulatory networks a new direction in the diagnostics and therapeutics for asthma.

## Figures and Tables

**Table:** 

ncRNAs:	Non-coding RNAs
miRNAs:	MicroRNAs
lncRNAs:	Long non-coding RNAs
circRNAs:	Circular RNAs
mRNA:	Messenger RNA
ceRNA:	Competing endogenous RNA
ASMCs:	Airway smooth muscle cells
Th2 cells:	T helper 2 cells
IgE:	Immunogloubulin E
MRE:	MiRNA response element
TGF-β:	Transforming growth factor-β
CTLA-4:	Cytotoxic T lymphocyte-associated protein 4
TNF-α:	Tumor necrosis factor-alpha
OVA:	Ovalbumin
PPM1A:	Protein phosphatase, Mg2+/Mn2+-dependent 1A
ORMDL3:	Orosomucoid 1-like 3
BALF:	Bronchoalveolar lavage fluid
CCR3:	CC chemokine receptor 3
IgE:	Immunoglobulin E
Eotaxin-1:	Eosinophil chemokine-1
SELP:	P-selectin
TSLP:	Thymic stromal lymphopoietin
NF-κB:	nuclear factor-kappa
BTLRs:	Toll-like receptors
IFN-g:	Interferon gamma
GSK-3β:	Glycogen synthase kinase-3β
KLF4:	Krüppel-like factor 4
SFRP4:	Secreted frizzled-related proteins4
Wnt:	Wingless/Integrase-1
EGFR:	Epidermal growth factor receptor
ATG5:	Autophagy-associated protein 5
NHBE:	Normal human bronchial epithelial
LPS:	Lipopolysaccharide
LncRNA GAS5:	LncRNA growth arrest-specificl 5
RMRP:	RNA component of mitochondrial RNA processing endoribonuclease
CCL2:	C-C motif ligand 2
PDGF-BB:	Platelet-derived growth factor-BB
IPSC:	Induced pluripotent stem cell
MSCs:	Mesenchymal stem cells
DUSP6:	Dual-specificity phosphatase 6
CSE:	CS extract
ICF:	Inducible costimulatory factors
VEGF:	Vascular endothelial growth factor
ADSCs:	Adipose-derived stem cells
STIM1:	Stromal interaction molecule 1
